# Prone position in intubated, mechanically ventilated patients with COVID-19: a multi-centric study of more than 1000 patients

**DOI:** 10.1186/s13054-021-03552-2

**Published:** 2021-04-06

**Authors:** Thomas Langer, Matteo Brioni, Amedeo Guzzardella, Eleonora Carlesso, Luca Cabrini, Gianpaolo Castelli, Francesca Dalla Corte, Edoardo De Robertis, Martina Favarato, Andrea Forastieri, Clarissa Forlini, Massimo Girardis, Domenico Luca Grieco, Lucia Mirabella, Valentina Noseda, Paola Previtali, Alessandro Protti, Roberto Rona, Francesca Tardini, Tommaso Tonetti, Fabio Zannoni, Massimo Antonelli, Giuseppe Foti, Marco Ranieri, Antonio Pesenti, Roberto Fumagalli, Giacomo Grasselli, PRONA-COVID Group, Angela Berselli, Tiziana Bove, Plinio Calligari, Irene Coloretti, Antonio Coluccello, Elena Costantini, Vito Fanelli, Giuseppe Gagliardi, Federico Longhini, Federica Mariani, Annalisa Mascarello, Luca Menga, Irene Ottaviani, Daniela Pasero, Matteo Pedeferri, Angelo Pezzi, Giuseppe Servillo, Paolo Severgnini, Savino Spadaro, Vanessa Zambelli

**Affiliations:** 1 Department of Medicine and Surgery, University of Milan-Bicocca, Monza, Italy; 2 Department of Anesthesia and Intensive Care Medicine, Niguarda Ca’ Granda, Milan, Italy; 3 Department of Anesthesia, Critical Care and Emergency, Fondazione IRCCS Ca’ Granda Ospedale Maggiore Policlinico, Via Francesco Sforza 35, 20122 Milan, Italy; 4 Department of Pathophysiology and Transplantation, University of Milan, Milan, Italy; 5 Ospedale di Circolo e Fondazione Macchi, Università degli studi dell’Insubria, Varese, Italy; 6 Department of Anesthesiology and Intensive Care, ASST Mantova–Ospedale Carlo Poma, Mantova, Italy; 7 Department of Biomedical Sciences, Humanitas University, Pieve Emanuele, MI Italy; 8 Division of Anaesthesia, Analgesia and Intensive Care, Department of Medicine and Surgery, University of Perugia, Perugia, Italy; 9 Department of Anesthesia and Intensive Care, A. Manzoni Hospital, ASST Lecco, Lecco, Italy; 10 Department of Anesthesia and Intensive Care, University Hospital of Modena, Modena, Italy; 11 Department of Anesthesiology, Intensive Care and Emergency Medicine, Fondazione Policlinico Universitario A. Gemelli IRCCS, Rome, Italy; 12 Sacred Heart Catholic University, Rome, Italy; 13 Department of Medical and Surgical Sciences, Intensive Care Unit, University of Foggia, Foggia, Italy; 14 Department of Anaesthesia and Intensive Care, Humanitas Clinical and Research Center-IRCCS, Rozzano, MI Italy; 15 Department of Anesthesia and Intensive Care Medicine, San Gerardo Hospital ASST Monza, Monza, Italy; 16 Anesthesia and Intensive Care Medicine, Policlinico di Sant’Orsola, Alma Mater Studiorum University of Bologna, Bologna, Italy; 17 Department of Medicine, University of Udine, Udine, Italy; 18 Plinio Calligari Ospedale Magalini Villafranca, Verona, Italy; 19 Department of Anesthesiology and Intensive Care, ASST Cremona - Ospedale di Cremona, Cremona, Italy; 20 Department of Surgical Science, Division of Anesthesia and Critical Care Medicine, University of Turin, AOU Città della Salute e della Scienza di Torino, Turin, Italy; 21 UOC Anestesia e Rianimazione, AULSS 5 Polesana, Rovigo, Italy; 22 Anesthesia and Intensive Care, Department of Medical and Surgical Sciences, “Magna Graecia” University, Catanzaro, Italy; 23 Department Morphology, Surgery and Experimental Medicine, Section of Intensive Care, University of Ferrara, Ferrara, Italy; 24 Anesthesia and Intensive Care Unit, AOU Sassari, Sassari, Italy; 25 Department of Medical, Surgical and Sperimental Science, University of Sassari, Sassari, Italy; 26 U.O.C. Anstesia e Rianimazione, Ospedale L. Mandic Merate, ASST Lecco, Merate, Italy; 27 ASST Nord Milano, Ospedale “E. Bassini”, San Giovanni, Italy; 28 Dipartimento di Neuroscienze, scienze riproduttive e odontostomatologiche, Università degli studi di Napoli Federico II, Naples, Italy

**Keywords:** COVID-19, Mechanical ventilation, Prone positioning, Refractory hypoxemia

## Abstract

**Background:**

Limited data are available on the use of prone position in intubated, invasively ventilated patients with Coronavirus disease-19 (COVID-19). Aim of this study is to investigate the use and effect of prone position in this population during the first 2020 pandemic wave.

**Methods:**

Retrospective, multicentre, national cohort study conducted between February 24 and June 14, 2020, in 24 Italian Intensive Care Units (ICU) on adult patients needing invasive mechanical ventilation for respiratory failure caused by COVID-19. Clinical data were collected on the day of ICU admission. Information regarding the use of prone position was collected daily. Follow-up for patient outcomes was performed on July 15, 2020. The respiratory effects of the *first* prone position were studied in a subset of 78 patients. Patients were classified as *Oxygen Responders* if the PaO_2_/FiO_2_ ratio increased ≥ 20 mmHg during prone position and as *Carbon Dioxide Responders* if the ventilatory ratio was reduced during prone position.

**Results:**

Of 1057 included patients, mild, moderate and severe ARDS was present in 15, 50 and 35% of patients, respectively, and had a resulting mortality of 25, 33 and 41%. Prone position was applied in 61% of the patients. Patients placed prone had a more severe disease and died significantly more (45% vs. 33%, *p* < 0.001). Overall, prone position induced a significant increase in PaO_2_/FiO_2_ ratio, while no change in respiratory system compliance or ventilatory ratio was observed. Seventy-eight % of the subset of 78 patients were *Oxygen Responders*. Non-Responders had a more severe respiratory failure and died more often in the ICU (65% vs*.* 38%, *p* = 0.047). Forty-seven % of patients were defined as *Carbon Dioxide Responders*. These patients were older and had more comorbidities; however, no difference in terms of ICU mortality was observed (51% vs*.* 37%, *p* = 0.189 for *Carbon Dioxide Responders* and *Non-Responders*, respectively).

**Conclusions:**

During the COVID-19 pandemic, prone position has been widely adopted to treat mechanically ventilated patients with respiratory failure. The majority of patients improved their oxygenation during prone position, most likely due to a better ventilation perfusion matching.

*Trial registration*: clinicaltrials.gov number: NCT04388670

**Supplementary Information:**

The online version contains supplementary material available at 10.1186/s13054-021-03552-2.

## Background

At the end of 2019, an outbreak of pneumonia of unknown etiology started from Wuhan, Hubei, China and subsequently spread worldwide. Italy was hit at the end of February 2020 and, as of the end of July 2020, more than 250,000 infections and more than 35,000 deaths had been reported [1]. A novel beta-coronavirus, named Severe Acute Respiratory Syndrome Coronavirus 2 (SARS-COV-2), was identified as the cause of the epidemic [2], and the resulting disease was called Coronavirus Disease 2019 (COVID-19). COVID-19 has a broad spectrum of clinical presentations, ranging from asymptomatic to extremely severe forms. A significant proportion of infected subjects develops the acute respiratory distress syndrome (ARDS) [3, 4] and requires invasive mechanical ventilation and admission to an intensive care unit (ICU) [4, 5].

In patients developing refractory hypoxemia despite invasive mechanical ventilation, the application of rescue therapies such as extracorporeal gas exchange, inhaled nitric oxide and prone positioning is frequently required [6]. Previous experience in patients with moderate-to-severe ARDS from different causes showed that early application of prone position is associated with a significant survival benefit [7–9]. In patients with ARDS, prone positioning should favour the re-expansion of collapsed lung parenchyma in dorsal lung regions, and reduction in aeration in ventral ones, leading both to lung recruitment and more homogenous lung aeration. While distribution of ventilation is certainly influenced by the postural change, lung perfusion is usually considered less dependent on gravity [10, 11]. Nevertheless, the net effect is usually a better ventilation-perfusion matching in prone position, resulting in improved gas exchange. Moreover, the more homogenous distribution of ventilation should reduce the risk of ventilator-induced lung injury.

Given the high number of COVID-19 patients with respiratory failure treated outside the ICU, there has been an increasing interest in the use of prone position in awake, spontaneously breathing patients [12–16]. On the contrary, limited data are available on the use of prone position in intubated, invasively ventilated patients [17, 18].

Aims of the present study are: (1) to describe the frequency of use of prone positioning and the clinical characteristics and outcomes of patients undergoing prone positioning in a large cohort of critically ill, mechanically ventilated patients with COVID-19; and (2) to describe, in a subgroup of patients, the pathophysiological effects of prone positioning.

## Methods

### Study design

This Italian multicentre, retrospective study of prospectively collected data was approved by the Ethical Committees of all participating centres (Promoting Centre’s Ethical Committee: Comitato Etico Milano Area 2; protocol: 0008489; date of approval: March 20, 2020) and registered at clinicaltrials.gov (NCT04388670). The need for informed consent from individual patients was waived owing to the retrospective nature of the study.

All patients admitted between February 22, 2020, and June 14, 2020, inclusive for those days, to the COVID-19 ICUs of 24 Italian hospitals (see Additional file 1: Table E1 for the complete list) were screened for eligibility. Laboratory-confirmed SARS-CoV-2 infection, (i.e. positive result of real-time reverse transcriptase–polymerase chain reaction assay of nasal and pharyngeal swabs), and ICU admission for ARDS, defined by the Berlin criteria [19], constituted the inclusion criteria. Exclusion criteria were age < 18 years, patients treated exclusively with non-invasive respiratory support and missing clinical data regarding the use of prone position. Clinical management (including mechanical ventilation setting and pharmacological therapies) followed the local treatment guidelines of each centre. The choice to position patients prone was at discretion of the attending physician.

**Table 1:** Patients' characteristics at admission in ICU and outcome

Variables	Overall (*n* = 1057)	Non-proned (*n* = 409, 39%)	Proned (*n* = 648, 61%)	*p* value
Males, no. (%)	831 (79)	317 (78)	514 (79)	0.483
Age (years)	63 (55–69)	63 (55–69)	63 (55–69)	0.773
BMI (kg/m^2^)	28 (25–31)	27 (25–31)	28 (25–31)	0.023
SOFA score	4 (3–5)	4 (3–4)	4 (3–5)	< 0.001
APACHE II score	10 (7–13)	9 (7–13)	10 (8–13)	0.013
Intubated, no. (%)^a^	892 (84)	351 (86)	541 (84)	0.309
Respiratory Rate (breaths/min)^b^	20 (18–25)	20 (16–24)	20 (18–25)	< 0.001
FiO_2_ (%)^b^	70 (60–90)	60 (50–80)	80 (60–90)	< 0.001
PEEP (cmH_2_O)^b^	12 (10–14)	12 (10–14)	12 (10–14)	< 0.001
PaO_2_/F_I_O_2_ ratio^b^	120 (88–173)	145 (107–197)	108 (81–148)	< 0.001
ARDS severity, no. (%)^b^				
Mild	128 (15)	76 (23)	52 (10)	< 0.001
Moderate	426 (50)	183 (56)	243 (46)	
Severe	298 (35)	69 (21)	229 (44)	
Tidal volume/PBW (mL/kg)^b^	7.0 (6.3–7.8)	7.1 (6.3–7.9)	7.0 (6.2–7.8)	0.140
Plateau pressure (cmH_2_O)^b^	24 (22–27)	24 (21–26)	25 (22–28)	< 0.001
Driving pressure (cmH_2_O)^b^	12 (9–14)	12 (9–13)	12 (9–14)	0.120
Respiratory system compliance (mL/cmH_2_O)^b^	40 (33–50)	42 (35–50)	38 (32–50)	0.035
pH	7.39 (7.32–7.46)	7.40 (7.33–7.46)	7.39 (7.31–7.45)	0.220
PaO_2_ (mmHg)	80 (67–101)	86 (70–108)	77 (65–97)	< 0.001
PaCO_2_ (mmHg)	43 (36–52)	43 (37–51)	43 (36–53)	0.710
Ventilatory ratio^b^	1.7 (1.4–2.2)	1.7 (1.3–2.1)	1.8 (1.4–2.2)	0.061
LDH (units/L)	479 (359–640)	424 (324–593)	507 (392–667)	< 0.001
D-dimer (ng/mL)	1492 (608–4602)	1190 (520–3470)	1730 (690–6576)	0.001
Ferritine (ng/mL)	1408 (811–2399)	1214 (668–1903)	1552 (1031–2491)	0.003
ICU mortality, no. (%)	374 (36)	112 (28)	262 (41)	< 0.001
Hospital mortality, no. (%)	405 (41)	127 (33)	278 (45)	< 0.001
ICU LOS (days)	15 (9–25)	12 (7–21)	16 (11–28)	< 0.001
Hospital LOS (days)	29 (17–46)	26 (16–40)	30 (17–49)	0.008
Mechanical ventilation (days)	14 (8–26)	10 (6–19)	16 (10–30)	< 0.001

Data are expressed either as median [interquartile range] or as frequency (percentage)

BMI = Body Mass Index; SOFA = Sequential Organ Failure Assessment; APACHE II = Acute Physiologic Assessment and Chronic Health Evaluation II; FiO_2_ = Inspired fraction of oxygen; PEEP = Positive End-Expiratory Pressure; PBW: Predicted Body Weight; PaO_2_: partial pressure of oxygen in arterial blood; PaCO_2_: partial pressure of carbon dioxide in arterial blood; LDH: lactate dehydrogenase; LOS = Length of stay

^a^Patients intubated same day of ICU admission

^b^Values refer to patients intubated on the same day of ICU admission

The population of patients included in the analysis was subdivided in two groups according to the use of prone positioning: (1) *PP group:* patients who were turned prone at least once during their ICU stay; and (2) *SP group*: patients always treated in the supine position.

### Data collection

An electronic case report form (REDCap electronic data capture tools) hosted at IRCCS Ca’ Granda Ospedale Maggiore Policlinico was used for data collection. An extensive set of information regarding demographic and anthropometric data, comorbidities [20] and clinical data (severity scores [21–23]**,** vital signs, type of respiratory support, use of prone positioning, respiratory parameters, laboratory tests including blood gas analysis) was collected on the day of admission to the ICU. Relevant clinical and laboratory data, including information regarding the use of prone positioning in the prior 24 h, were then collected daily until ICU discharge or patient death.

Finally, the following patient outcomes were recorded: ICU and hospital survival, ICU and hospital length of stay (LOS), duration of invasive mechanical ventilation. The final date of follow-up for patient outcomes were July 15, 2020.

### Effect of prone positioning on respiratory mechanics and gas exchange

To assess the physiologic effects of pronation, a subgroup of 78 patients who underwent prone positioning in two of the participating hospitals (Grande Ospedale Metropolitano Niguarda and Fondazione IRCCS Ca’ Granda Ospedale Maggiore Policlinico, both in Milan) was investigated at three different time points: (1) prior to the first pronation (Baseline); (2) during the last hour of the first session of prone ventilation (Prone); and (3) within 4 h after turning the patients back to supine position (Supine). At each time-point, end-inspiratory and end-expiratory airway occlusion manoeuvres were performed and arterial blood gases analyzed to obtain the following variables: compliance of the respiratory system (Crs, calculated as the ratio between tidal volume and airway driving pressure); ratio between partial pressure of oxygen (PaO_2_) and inspired fraction of oxygen (FiO_2_),—PaO_2_/FiO_2_ ratio; corrected minute ventilation [24] and ventilatory ratio [25]. Patients were defined as “O_2_*-Responders”*, if they had an increase in the PaO_2_/FiO_2_ ratio of ≥ 20 mmHg during prone ventilation as compared to baseline values in supine position [26, 27]. Moreover, patients were defined as *Responders* in terms of carbon dioxide (CO_2_) clearance, *“CO*_*2*_-*Responders”*, if their ventilatory ratio was reduced during prone ventilation, as compared to baseline values in supine position, i.e. if the difference between ventilatory ratio in prone position and ventilatory ratio at baseline (∆VR) was < 0.

### Statistical analysis

Continuous variables are presented as mean with standard deviation (SD) or median and interquartile range (IQR). Categorical variables are expressed as frequencies (percentages).

Mann–Whitney rank sum test was used to compare nonparametric continuous variables between study groups. *χ*^2^ or Fisher exact test was used for categorical variables, as appropriate.

Differences among time-points were tested by one-way ANOVA for repeated measures or Friedman Repeated Measures Analysis of Variance on Ranks, as appropriate. Pairwise multiple comparisons were tested using Tukey’s test. Differences among tertiles of pre-pronation driving pressure were tested by one-way ANOVA on ranks. Pairwise multiple comparisons were tested using Dunn's Method. All statistical tests were 2-tailed, and statistical significance was defined as a *p* value below 0.05. Analyses were performed using SAS 9.4 (SAS Institute Inc., Cary, NC, USA), STATA computer software, version 16.0 (StataCorp LLC) and SigmaPlot 12.0 (Systat Software Inc., San Jose, CA).

## Results

One thousand three hundred twenty-six patients fulfilled the inclusion criteria. After exclusions (one patient aged < 18 years, 123 patients with missing information regarding the use of prone position and 145 patients who were never intubated), 1057 patients were analyzed (Flowchart reported in Additional file 1: Fig. E1).

**Fig. 1:**
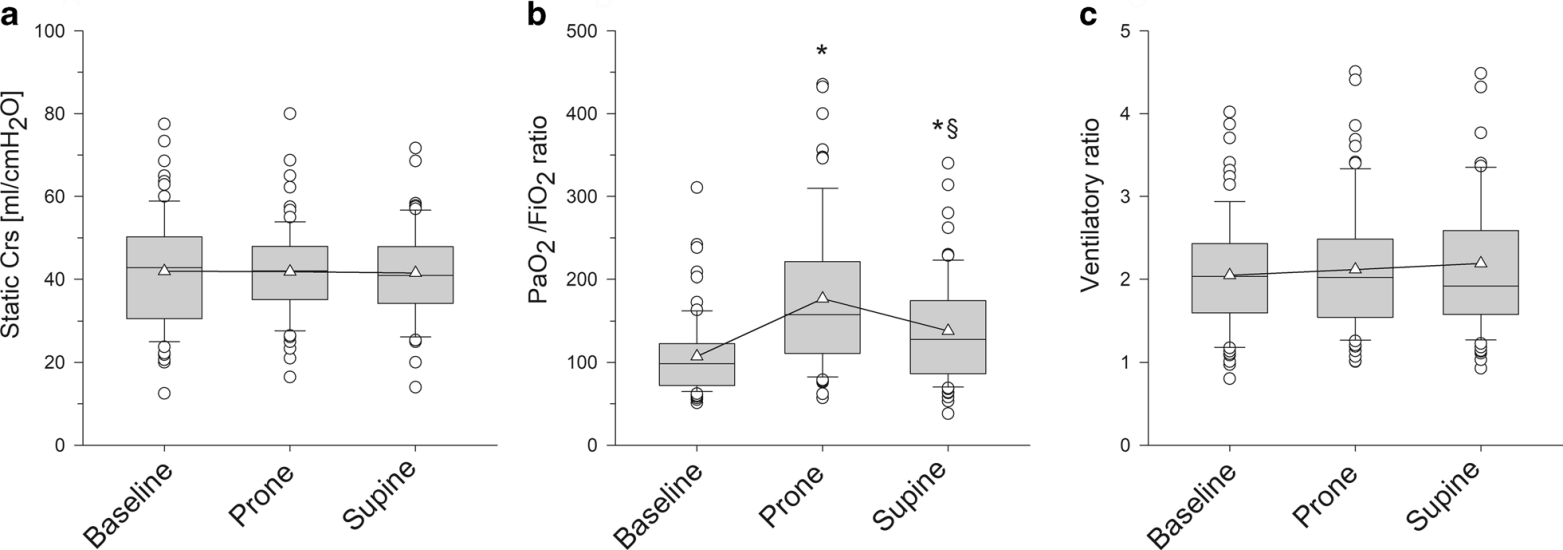
Physiological parameters’ changes during the first session of prone positioning

Table 1 summarizes the patients’ demographic and clinical characteristics at ICU admission and their clinical outcomes. Additional information is reported in Additional file 1, Table E2. Most patients were male (79%), median age was 63 [55–69] years, and median body mass index was 28 [25–31] kg/m^2^. Median SAPS II and SOFA score at ICU admission were 36 [30–44] and 4 [3–4], respectively. Eighty-four % of patients were intubated and mechanically ventilated at ICU admission or during the first day in ICU. ARDS severity was mild in 15%, moderate in 50% and severe in 35% of the cases. Median PaO_2_/FiO_2_ ratio, respiratory rate, tidal volume/predicted body weight and plateau airway pressure of mechanically ventilated patients were 120 mmHg [88–173], 20 [18–25] breaths/min, 7.0 [6.3–7.8] mL/kg and 24 [22–27] cmH_2_O, respectively. As of July 15, 2020, 677 (64%) patients had been discharged from the ICU and 374 (36%) had died (6 missing data). Mortality increased significantly with increasing severity of ARDS (25, 33, 41%, *p* = 0.004, for mild, moderate and severe ARDS, respectively). The median ICU length of stay was 16 [10–28] days for patients discharged from the ICU, and 12 [6–20] days for those who died in the ICU.

**Table 2:** Physiologic variables before, during and after prone positioning (*n* = 78)

Variables	Baseline	Prone	Supine	*p* value
PEEP (cmH_2_O)	14 (12–15)	14 (12–15)	14 (12–15)	0.679
FiO_2_ (%)	70 (60–90)	60 (50–70)^a^	60 (45–80)^a^	< 0.001
Tidal volume/PBW (mL/kg)	6.8 (6.1–7.6)	6.7 (6.2–7.3)	6.8 (6.2–7.4)	0.619
Driving pressure (cmH_2_O)	11 (10–14)	11 (10–14)	11 (9–14)	0.147
Plateau pressure (cmH_2_O)	25 (22–28)	24 (23–27)	24 (23–28)	0.324
Respiratory rate (breaths/min)	20 (18–22)	22 (20–24)^a^	22 (20–24)^a^	< 0.001
Minute ventilation (L/min)	9.4 (7.7–11.0)	9.6 (8.3–11.2)	9.9 (8.2–11.2)	0.052
Corrected minute ventilation (L/min)	12.6 (9.8–15.2)	12.6 (9.5–15.7)	12.2 (10.3–14.9)	0.881
PaCO_2_ (mmHg)	53 (45–60)	53 (43–59)	52 (46–60)	0.302
Respiratory system Compliance (mL/cmH_2_O)	43 (31–50)	42 (35–48)	41 (34–48)	0.943
PaO_2_/FiO_2_ ratio	98 (72–121)	158 (112–220)^a^	128 (87–174)^a,b^	< 0.001
Ventilatory ratio	2.0 (1.6–2.4)	2.0 (1.5–2.5)	1.9 (1.6–2.5)	0.881

Data are expressed as median (interquartile range)

PEEP = Positive End-Expiratory Pressure; FiO_2_ = fraction of inspired oxygen; PBW = Predicted Body Weight; PaCO_2_ = partial pressure of carbon dioxide in arterial blood

^a^*p* < 0.05 versus baseline

^b^*p* < 0.05 versus prone

### Use of prone positioning and differences between pronated and non-pronated patients

Six-hundred and forty-eight patients (61% of the overall population) were placed in prone position at least once during their stay in the ICU (PP Group), while 409 patients (39% of the overall population) were always treated in the supine position (SP Group). The frequency of use of prone positioning increased with ARDS severity (52/128 (44%), 243/426 (57%) and 229/298 (77%), *p* < 0.001, in mild, moderate and severe ARDS, respectively). Prone positioning was first applied 2 [1–4] days after ICU admission, and a median of 3 [1–4] pronation sessions per patient was performed.

Table 1 outlines the principal differences between the two groups (additional information is summarized in Additional file 1, Table E2). No difference in comorbidities was observed (Charlson Comorbidity Index 2 [1–3] vs*.* 2 [1–3], *p* = 0.165). Patients in the PP group had significantly more severe respiratory disease, as suggested by a higher percentage of severe ARDS (44% vs. 21%, *p* < 0.001) and a lower percentage of mild ARDS (10 vs. 23%, *p* < 0.001). Respiratory rate, positive end-expiratory pressure (PEEP), FiO_2_ and Plateau pressure were significantly higher, while respiratory system compliance, PaO_2_/FiO_2_ ratio and arterial pH at ICU admission were significantly lower in the PP group. In addition, biochemical markers of inflammation and disease severity, such as LDH, D-dimers and ferritin, were consistently higher in patients of the PP Group. Patients of the PP group had higher severity scores: SOFA (4 [3–5] vs*.* 4 [3–4], *p* < 0.001) and APACHE II scores (10 [8–13] vs*.* 9 [7–13], *p* < 0.001). Finally, ICU mortality and length of stay, length of mechanical ventilation and hospital mortality and length of stay were all significantly worse in patients in the PP group.

### Physiological effects of prone position

In the subgroup of 78 patients, median duration of the first pronation was 18.5 [16–22] hours. Respiratory system compliance did not change significantly with the change in body position (Fig. 1a). Similarly, on average, prone positioning had no significant effect on ventilatory ratio (Fig. 1c). Overall, prone positioning led to a significant increase in PaO_2_/FiO_2_ ratio, which was followed by a subsequent significant decrease with re-supination (Fig. 1b). On average, PaO_2_/FiO_2_ ratio after re-supination remained significantly higher as compared to baseline values. Table 2 summarizes the physiologic variables at the three different time points selected for the analysis.

### O_2_-responders versus O_2_-non-responders

**Fig. 2:**
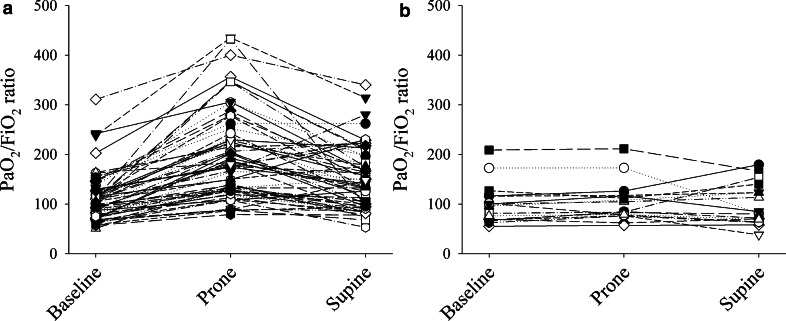
Individual variations in PaO_2_/FiO_2_ ratio in Responders and Non-Responders during the first session of prone positioning

Sixty-one out of 78 patients (78%) had an increase in PaO_2_/FiO_2_ ratio ≥ 20 mmHg (median increase 68 [42–117] mmHg) and where therefore defined as O_2_-*Responders*. Seventeen (22%) patients had an increase in PaO_2_/FiO_2_ ratio < 20 mmHg (median variation 3 [1–12] mmHg) and were therefore classified as *O*_*2*_*-Non-Responders*. Individual variations in PaO_2_/FiO_2_ ratio due to the change in body position in *O*_*2*_*-Responders* and *O*_*2*_-*Non-Responders* are reported in Fig. 2a, b, respectively. Table 3 summarizes the differences between *O*_*2*_-*Responders* and *O*_*2*_*-Non-Responders* (for additional information see Additional file 1, Table E3). Demographics, comorbidities and admission severity scores were similar between *O*_*2*_*-Responders* and *O*_*2*_-*Non-Responders*.

**Table 3:** Patients' characteristics at admission in ICU and outcome divided by O_2_-Responders versus O_2_-Non-Responders

Variables	Overall (*n* = 78)	O_2_-non-responders (*n* = 17, 22%)	O_2_-responders (*n* = 61, 78%)	*p* value
Males, no. (%)	61 (78)	13 (77)	48 (79)	0.845
Age (years)	62 (51–68)	56 (51–66)	62 (52–68)	0.389
BMI (kg/m^2^)	27 (25–31)	26 (24–31)	27 (26–31)	0.670
SOFA score	4 (3–5)	4 (3–5)	4 (4, 5)	0.294
APACHE II score	10 (8–12)	11 (8–14)	10 (8–12)	0.620
Intubated, no. (%)^a^	75 (96)	16 (94)	59 (97)	0.622
Respiratory rate (breaths/min)^b^	20 (18–22)	19 (18–22)	20 (17–22)	0.813
FiO_2_ (%)^b^	75 (60–90)	80 (60–88)	70 (60–90)	0.839
PEEP (cmH_2_O)^b^	14 (12–15)	14 (11–14)	14 (12–15)	0.662
PaO_2_/F_I_O_2_ ratio^b^	111 (83–164)	99 (72–150)	114 (85–168)	0.419
ARDS severity, no. (%)^b^				
Mild	10 (13)	2 (13)	8 (14)	0.721
Moderate	34 (45)	6 (38)	28 (48)	
Severe	31 (41)	8 (50)	23 (39)	
Tidal volume/PBW (mL/kg)^b^	7.0 (6.4–7.8)	7.2 (6.2–7.9)	7.0 (6.4–7.8)	0.707
Plateau pressure (cmH_2_O)^b^	25 (22–27)	27 (24–28)	24 (22–27)	0.043
Driving pressure (cmH_2_O)^b^	12 (9–14)	14 (12–15)	12 (8–13)	0.022
Respiratory system compliance (mL/cmH_2_O)^b^	42 (32–53)	34 (30–45)	45 (34–56)	0.018
pH	7.36 (7.31–7.41)	7.33 (7.29–7.42)	7.37 (7.32–7.41)	0.244
PaO_2_ (mmHg)	78 (70–95)	76 (68–90)	80 (70–99)	0.443
PaCO_2_ (mmHg)	47 (40–56)	50 (39–58)	47 (41–55)	0.417
Ventilatory ratio	1.8 (1.4–2.0)	1.8 (1.4–2.5)	1.8 (1.4–2.0)	0.345
LDH (units/L)	414 (307–490)	418 (329–485)	400 (301–490)	0.620
ICU mortality, no. (%)	34 (44)	11 (65)	23 (38)	0.047
Hospital mortality, no. (%)	34 (44)	11 (65)	23 (38)	0.047
ICU LOS (days)	18 (11–34)	18 (12–47)	19 (11–33)	0.981
Hospital LOS (days)	36 (17–58)	32 (14–47)	39 (21–61)	0.295
Mechanical ventilation (days)	19 (11–35)	16 (11–35)	19 (11–35)	0.832

Data are expressed either as median (interquartile range) or as frequency (percentage)

BMI = Body Mass Index; SOFA = Sequential Organ Failure Assessment; APACHE II = Acute Physiologic Assessment and Chronic Health Evaluation II; FiO_2_ = Inspired fraction of oxygen; PEEP = Positive End-Expiratory Pressure; PBW: Predicted Body Weight; PaO_2_: partial pressure of oxygen in arterial blood; PaCO_2_: partial pressure of carbon dioxide in arterial blood; LDH: lactate dehydrogenase; LOS = Length of stay

^a^Patients intubated same day of ICU admission

^b^Values refer to patients intubated on the same day of ICU admission

Notably, at ICU admission, driving pressure (14 [12–15] vs*.* 12 [8–13] cmH_2_O, *p* = 0.022), plateau pressure (27 [24–28] vs*.* 24 [22–27] cmH_2_O, *p* = 0.043) and respiratory system compliance (34 [30–45] vs*.* 45 [34–56] mL/cmH_2_O, *p* = 0.018) were significantly different between *O*_*2*_*-Responders* and *O*_*2*_-*Non-Responders*. Moreover, prior to first pronation, baseline driving pressure (14 [11–16] vs*.* 11 [10–13] cmH_2_O, *p* = 0.036), respiratory rate (22 [20–24] vs*.* 20 [18–22] breaths per minute, *p* = 0.014), PaCO_2_ (58 [50–67] vs*.* 52 [45–60] mmHg, *p* = 0.092) and ventilatory ratio (2.2 [1.9–2.7] vs*.* 1.9 [1.6–2.2], *p* = 0.014) were higher in *O*_*2*_*-Non-Responders*, while Respiratory System Compliance (33 [26–45] vs. 44 [33–51] mL/cmH_2_O, *p* = 0.029), and pH (7.33 [7.31–7.38] vs*.* 7.37 [7.34–7.40], *p* = 0.041) were lower*.* When dividing the overall population in tertiles of pre-pronation driving pressure, a significantly different variation in PaO_2/_FiO_2_ ratio was observed, with patients with lower driving pressures having greater increase in PaO_2_/FiO_2_ ratio (Additional file 1: Fig. E2). ICU mortality (11/17, 65% vs. 23/61, 38%, *p* = 0.047) was higher in *O*_*2*_*-Non-Responders.* Similar results were observed using a 10–20% increase in PaO_2_/FiO_2_ ratio as cut off (8/11, 72% vs. 26/67, 39%, *p* = 0.035 at 10% increase in PaO_2_/FiO_2_ ratio and 9/15, 60% vs. 25/63, 40%, *p* = 0.154 at 20% increase in PaO_2_/FiO_2_ ratio).

### CO_2_-responders versus CO_2_-non responders

Thirty-seven out of 78 patients (47%) reduced their ventilatory ratio during prone position (median ∆VR − 0.21 [− 0.36 to − 0.10]) and where therefore defined as *CO*_*2*_*-Responders*. In 41 (53%) patients, the ventilatory ratio did not change or increased in prone position (median ∆VR 0.28 [0.09–0.54]): These patients were therefore defined as *CO*_*2*_*-Non Responders.* Differences between *Responders* and *Non-Responders* in terms of CO_2_ clearance are summarized in Additional file 1, Table E4. In summary, no differences were observed in the two populations, except for older age (65 [59–70] years vs*.* 56 [50–64] years, *p* = 0.005) and higher prevalence of hypertension (68% vs*.* 34%, *p* = 0.003) in *CO*_*2*_*-Non Responders*. ICU mortality did not differ between the two groups (19/37, 51% vs*.* 15/41, 37%, *p* = 0.189 in *CO*_*2*_*-Responders* and *CO*_*2*_*-Non-Responders*, respectively).

## Discussion

In this national, multicentre, retrospective observational study performed in the ICUs of 24 Italian hospitals during the first peak of the 2020 COVID-19 pandemic, we investigated the use of prone positioning in a cohort of 1057 critically ill, invasively ventilated patients with respiratory failure due to COVID-19. We also analyzed the pathophysiologic respiratory effects of this manoeuvre in a subset of 78 patients. A major finding of our study is that prone positioning was applied very frequently, significantly more often than previously reported in other populations of ARDS patients [28, 29]. Indeed, 61% of our patients underwent at least one pronation session during their ICU stay, as compared to 8% of the patients enrolled in the LUNG SAFE study. The frequency of use of prone positioning increased with increasing ARDS severity. Notably, 77% of COVID-19 patients with severe ARDS underwent prone positioning, as compared to the 16% of those with severe ARDS in the LUNG SAFE cohort. Of note, prone position was also frequently applied in patients with mild and moderate ARDS at ICU admission.

Changing body position from supine to prone (or vice versa) requires dedicated and experienced personnel. Moreover, the manoeuvre frequently requires incremental dosages of sedatives and muscle relaxants [30] and may lead to hemodynamic instability. In addition, it is associated with an increased risk of device displacement and pressure ulcers [31]. It is important to underline that in our study, the decision to turn the patients in prone position was at the discretion of the ICU team, i.e. there were no pre-specified criteria for the application of this rescue manoeuvre. Due the overwhelming number of critically ill patients requiring ICU admission, the ICU bed capacity of our hospitals had to be rapidly increased [32]. Therefore, many physicians and nurses usually working outside the ICU environment and even doctors from other specialities were recruited to allow the surge in ICU capacity. This of course reduced the expertise of the whole ICU staff. Our data clearly show that prone positioning was applied to patients with more severe disease, mainly as a rescue therapy (Table 1). Consequently, the worse clinical outcomes of patients undergoing prone positioning can be explained by the higher disease severity. However, given the retrospective nature of the study, we cannot draw any conclusions on the efficacy of prone position in terms of outcome.

Another important finding, resulting from the physiological sub-study, is that, on average, the PaO_2_/FiO_2_ ratio increased significantly from 98 [72–212] to 158 [112–220] mmHg, *p* < 0.001 (Fig. 1b) during the first pronation session. Moreover, while the PaO_2_/FiO_2_ ratio dropped with re-supination, as previously observed [33, 34], values after re-supination remained significantly higher than baseline values (128 [87–174] vs. 98 [72–212], *p* < 0.05).

The findings of the physiologic sub-study (Table 2) thus suggest that the main mechanism inducing an improvement in oxygenation during the first pronation of COVID-19 patients with ARDS is the improvement of the ventilation-perfusion matching, possibly favoured by a redistribution of flow from dorsal to ventral lung areas. Indeed, the lack of improvement of respiratory system compliance with the change in body position (Fig. 1a) suggests that lung recruitment was not the major mechanism. This hypothesis is also suggested by the fact that patients with lower driving pressure/higher respiratory system compliance and thus higher lung volumes, had, on average, greater increases in PaO_2_/FiO_2_ ratio (Fig. E2). We observed a modest, though significant increase in set respiratory rate and a resulting trend toward higher minute ventilations during prone positioning (Table 2). However, we did not observe a significant variation of the ventilatory ratio, a proxy of dead space and efficiency in CO_2_ removal (Fig. 1c). Taken together, these results suggest that CO_2_ production somehow increased during prone position, requiring an increase in minute ventilation to maintain stable PaCO_2_ values.

We used an increase in PaO_2_/FiO_2_ ratio during pronation of at least 20 mmHg as cut-off to define the response to prone position in terms of oxygenation. Using this definition, 78% of the studied patients were considered “*O*_*2*_*-Responders”.* There are no universally applied criteria to define the response to prone position, however, when looking at the literature using the same cut-off [26, 27], the percentage of patients with COVID-19-induced ARDS that responded to prone position seems similar to the percentage of the “general” ARDS population [26].

When analyzing the differences between *O*_*2*_*-Responders* and *Non-Responders*, we observed that, despite similar comorbidities and baseline severity scores, respiratory failure was on average more severe in *O*_*2*_*-Non-Responders* (Table 3). Indeed, *Non-Responders* had higher driving pressure and ventilatory ratio, suggesting a higher extension of lung dysfunction and a lower efficiency of gas exchange. In the ARDS literature, several studies did not find a different mortality between *Responders* and *Non-Responders* in terms of oxygenation [26, 27], while a recent study performed on ARDS, non-COVID patients, suggested that improved oxygenation after prone positioning might be a predictor of survival [35]. Also in our study performed in COVID-19 ARDS patients, we found that the mortality of *O*_*2*_*-Non-Responders* was significantly higher as compared to *Responders* (65% vs*.* 38%, *p* = 0.039)*.*

In order to evaluate the response to pronation in terms of CO_2_ clearance, we analyzed the variations in ventilatory ratio. Also in this case, there is no universally applied criteria to define *CO*_*2*_*-Respond*ers, and several cut-offs of absolute changes in partial pressure of CO_2_ (PCO_2_) during prone position have been previously used [26, 36–38]. The variation in PCO_2_ is used as a proxy of the efficiency of the system to eliminate CO_2_, i.e. pulmonary dead space fraction. Of course, this proxy can be evaluated only if the ventilatory settings do not change during prone position and, ideally, if the CO_2_ production is stable. We recorded a significant increase in respiratory rate and thus minute ventilation during prone position and therefore could not use the variation in PCO_2_ as a proxy of dead space variation. We therefore used a variation in the ventilatory ratio to differentiate between *CO*_*2*_*-Responders* and *Non-Responders*. In this exploratory analysis, *CO*_*2*_*-Non-Responders* were found to be older and with more comorbidities; however, no significant difference in outcome was observed.

### Limitations

The retrospective observational nature of the study is a clear limitation of our study. As already discussed, the decision to place the patient in prone position was at discretion of the attending physicians and the general clinical patient management was not standardized among centres. The comparison between the two groups gives therefore useful information about the decision-making process of Italian doctors caring for severely ill COVID-patients during the first wave of the 2020 COVID pandemic. On the contrary, the comparison does not provide information about the efficacy of pronation in terms of outcome. In addition, we have not collected information regarding complications related to prone positioning. A certain rate of complications usually occurs during prone position. It is conceivable that the rate might be higher in the specific context of a pandemic surge. Regarding the physiologic sub-study, the absence of information of partitioned respiratory mechanics is certainly a limitation. Nevertheless, the fact that the respiratory system compliance did not change in the 3 time-points suggests that lung recruitment did not play a significant role during the first pronation. Moreover, the limited number of patients included in the physiologic sub-study limits the soundness of the observed differences between *Responders* and *Non-Responders*, both in terms of oxygenation and carbon dioxide clearance.

## Conclusions

During the most intense months of the first wave of 2020 COVID-19 pandemic in Italy, critically ill, intubated and mechanically ventilated patients with ARDS were frequently placed in prone position. The more severe the respiratory failure, the more frequent the use of this rescue therapy. Placing the patients in prone position has the main purpose of reducing the injurious effects of mechanical ventilation. In addition, it is a cheap and effective manoeuvre, able to improve oxygenation in the vast majority of patients with respiratory failure due to COVID-19. In this population, the main mechanisms responsible for the improved oxygenation seems to be the improvement of the ventilation/perfusion matching.

## Supplementary Information

**Additional file 1.** This additional file contains three additional tables and 1 additional figure.

## Data Availability

The dataset used and/or analysed during the current study are available from the corresponding author on reasonable request.

## Abbreviations

APACHE II: Acute Physiology and Chronic Health Disease Classification System II
ARDS: Acute respiratory distress syndrome
COVID-19: Coronavirus Disease 2019
FiO_2_: Inspired fraction of oxygen
ICU: Intensive Care Unit
IQR: Interquartile Range
LDH: Lactate Dehydrogenase
LOS: Length of Stay
PaCO_2_: Partial pressure of carbon dioxide in arterial blood
PaO_2_: Partial pressure of oxygen in arterial blood
PEEP: Positive end-expiratory pressure
PP: Prone Position
SAPS II: Simplified Acute Physiology Score II
SARS-COV-2: Severe Acute Respiratory Syndrome Coronavirus 2
SOFA: Sequential Organ Failure Assessment
SP: Supine Position
SD: Standard Deviation
VR: Ventilatory ratio
