# Supplementary material for: Prone position in intubated, mechanically ventilated patients with COVID-19: a multi-centric study of more than 1000 patients
Source: Crit Care. 2021 Apr 6;25:128. doi: 10.1186/s13054-021-03552-2 (PMC8022297; doi:10.1186/s13054-021-03552-2)
Supplement: Supplementary file 1 — Additional file 1. This additional file contains three additional tables and 1 additional figure. [file 13054_2021_3552_MOESM1_ESM.docx]

**Prone position in intubated, mechanically ventilated patients**

**with COVID-19: a multi-centric study of more than 1000 patients.**

**Authors:** Thomas Langer, MD; Matteo Brioni, MD; Amedeo Guzzardella, MD; Eleonora Carlesso, MS; Luca Cabrini, MD; Gianpaolo Castelli, MD; Francesca Dalla Corte, MD; Edoardo De Robertis, PhD; Martina Favarato, MD; Andrea Forastieri, MD; Clarissa Forlini, MD; Massimo Girardis, MD; Domenico Luca Grieco, MD; Lucia Mirabella, MD; Valentina Noseda, MD; Paola Previtali, MD; Alessandro Protti, MD; Roberto Rona, MD; Francesca Tardini, MD; Tommaso Tonetti, MD; Fabio Zannoni, MD; Massimo Antonelli, MD; Giuseppe Foti, MD; Marco Ranieri, MD; Antonio Pesenti, MD; Roberto Fumagalli, MD; Giacomo Grasselli, MD; PRONA-COVID Group.

# **Additional File**

## **Additional Methods**

**Table E1. List of the 24 Italian Hospitals participating in the study.**

| **Hospital** | **City** |
| --- | --- |
| Fondazione IRCCS Cà Granda Ospedale Maggiore Policlinico | Milan |
| ASST Grande Ospedale Metropolitano Niguarda | Milan |
| ASST MONZA - Ospedale San Gerardo | Monza |
| AO Carlo Poma | Mantova |
| ASST dei Sette Laghi, Ospedale di Circolo e Fondazione Macchi | Varese |
| Humanitas Research Hospital | Milan |
| Ospedale San Giovanni Molinette | Torino |
| Azienda Ospedaliera – Universitaria di Bologna, Policlinico S. Orsola Malpighi | Bologna |
| ASST Lecco Ospedale di Merate | Merate |
| ASST Lecco - Ospedale 'A. Manzoni' | Lecco |
| ASST Nord Milano - Ospedale Edoardo Bassini - Cinisello Balsamo | Cinisello Balsamo |
| ASST Monza - Ospedale di Desio | Desio |
| AULSS 5 Polesana - Ospedale di Rovigo e Ospedale di Trecenta | Rovigo - Trecenta |
| AULSS 9 Scaligera - Ospedale Magalini di Villafranca | Verona |
| Azienda Ospedaliera di Perugia | Perugia |
| ASST Cremona | Cremona |
| Azienda Sanitaria Universitaria Friuli Centrale - Udine | Udine |
| Azienda Ospedaliero - Universitaria di Modena | Modena |
| Azienda Ospedaliera-Universitaria di Sassari | Sassari |
| Policlinico Universitario Fondazione Agostino Gemelli | Roma |
| Azienda Ospedaliera Mater Domini di Catanzaro | Catanzaro |
| Azienda Ospedaliera Universitaria Federico II | Napoli |
| Azienda Ospedaliera-Universitaria di Ferrara | Ferrara |
| Azienda Ospedaliera-Universitaria Foggia Ospedali riuniti | Foggia |

## **Additional Results**

**Figure E1. Flow chart**

## **
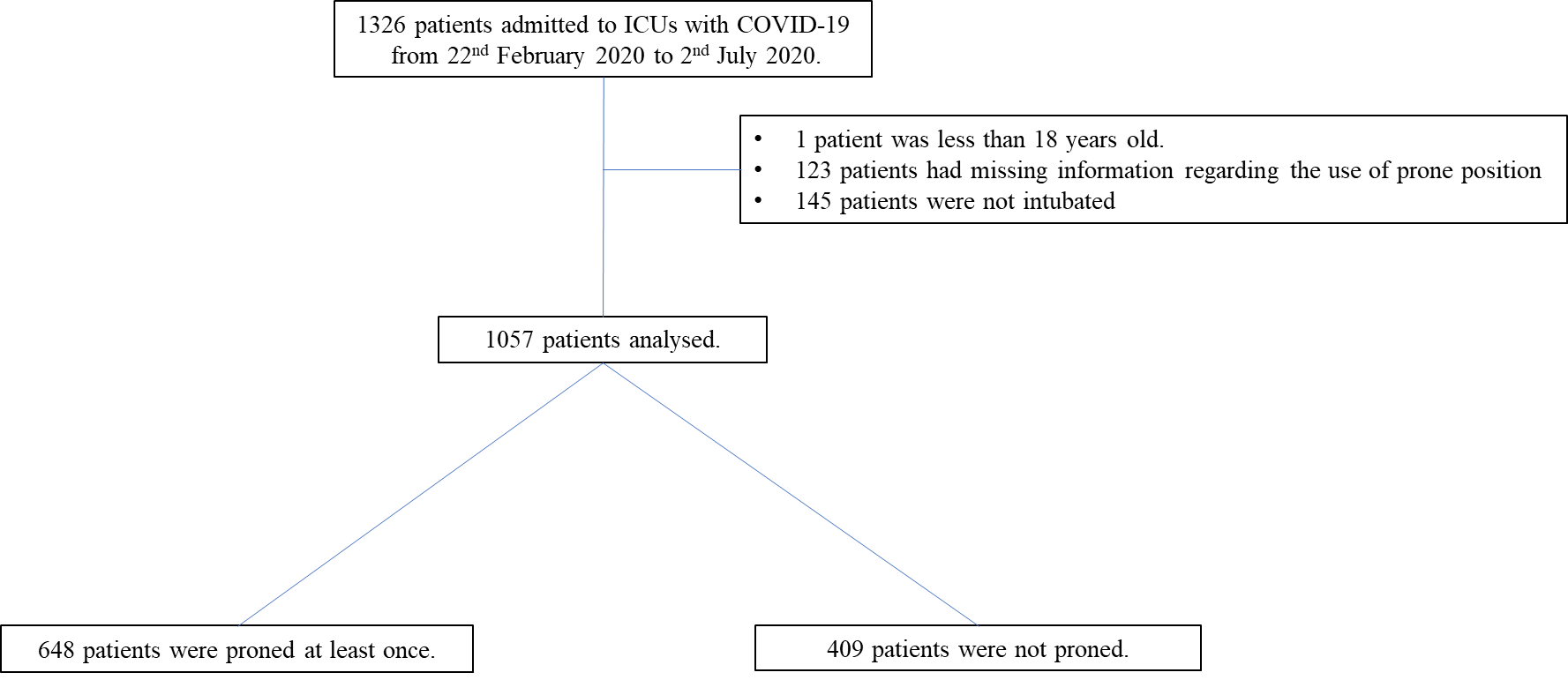
**

**Table E2. Complete patients' characteristics at admission in ICU and outcome**

| **Variables** | **Overall**  **(n = 1057)** | **Non-proned**  **(n = 409, 39%)** | **Proned**  **(n = 648, 61%)** | **p-value** |
| --- | --- | --- | --- | --- |
| **Males, no. (%)** | 831 (79) | 317 (78) | 514 (79) | 0.483 |
| **Age (years)** | 63 [55 - 69] | 63 [55 - 69] | 63 [55 - 69] | 0.773 |
| **BMI (kg/m^2^)** | 28 [25 - 31] | 27 [25 - 31] | 28 [25 - 31] | 0.023 |
| **Weight (kg)** | 80 [75 - 90] | 80 [72 - 90] | 82 [75 - 90] | 0.003 |
| **Height (cm)** | 172 [168 - 178] | 170 [166 - 178] | 173 [170 - 178] | 0.110 |
| **Charlson's Comorbidity Index** | 2 [1 - 3] | 2 [1 - 3] | 2 [1 - 3] | 0.165 |
| **Comorbidities** |  |  |  |  |
| Hypertension, no. (%) | 517 (49) | 198 (48) | 319 (49) | 0.796 |
| Others cardiovascular, no. (%) | 152 (14) | 73 (18) | 79 (12) | 0.011 |
| Diabetes, no. (%) | 193 (18) | 83 (20) | 110 (17) | 0.174 |
| Respiratory, no. (%) | 86 (8) | 30 (7) | 56 (9) | 0.449 |
| Malignancy, no. (%) | 72 (7) | 29 (7) | 43 (7) | 0.775 |
| Immunological, no. (%) | 53 (5) | 21 (5) | 32 (5) | 0.887 |
| Hepatic, no. (%) | 20 (2) | 10 (2) | 10 (2) | 0.295 |
| Renal, no. (%) | 22 (2) | 16 (4) | 6 (1) | 0.001 |
| Others, no. (%) | 415 (39) | 162 (40) | 253 (39) | 0.854 |
| None, no. (%) | 222 (21) | 79 (19) | 143 (22) | 0.285 |
| **SOFA Score** | 4 [3 - 5] | 4 [3 - 4] | 4 [3 - 5] | <0.001 |
| **SAPS II Score** | 36 [30 - 44] | 35 [29 - 44] | 36 [30 - 44] | 0.166 |
| **APACHE II Score** | 10 [7 - 13] | 9 [7 - 13] | 10 [8 - 13] | 0.013 |
| **Temperature (°C)** | 37 [36.3 - 37.8] | 36.9 [36.2 - 37.6] | 37 [36.3 - 37.8] | 0.145 |
| **Symptom onset to admission** | 10 [6 - 13] | 10 [6 - 14] | 10 [7 - 13] | 0.361 |
| **Symptom onset to intubation** | 10 [7 - 13] | 10 [7 - 14] | 10 [7 - 13] | 0.430 |
| **Presenting symptoms** |  |  |  |  |
| **Fever** | 945 (89) | 359 (88) | 586 (90) | 0.172 |
| **Dyspnea** | 596 (56) | 229 (56) | 367 (57) | 0.837 |
| **Cough** | 532 (50) | 194 (47) | 338 (52) | 0.134 |
| **Asthenia** | 118 (11) | 47 (11) | 71 (11) | 0.788 |
| **Myalgias** | 31 (3) | 13 (3) | 18 (3) | 0.707 |
| **Headache** | 22 (2) | 9 (2) | 13 (2) | 0.829 |
| **Respiratory Failure** | 58 (5) | 15 (4) | 43 (7) | 0.039 |
| **Oxygen delivery before IMV, no. (%)** | 1005 (96) | 381 (94) | 624 (97) | 0.013 |
| **Oxygen delivery time before IMV (hours)** | 48 [24 - 96] | 48 [24 - 96] | 48 [24 - 96] | 0.476 |
| **Intubated, no. (%) ^a^** | 892 (84.39) | 351 (85.82) | 541 (83.49) | 0.309 |
| **Respiratory Rate (breaths/min) ^b^** | 20 [18 - 25] | 20 [16 - 24] | 20 [18 - 25] | <0.001 |
| **FiO_2_ (%) ^b^** | 70 [60 - 90] | 60 [50 - 80] | 80 [60 - 90] | <0.001 |
| **PEEP (cmH_2_O) ^b^** | 12 [10 - 14] | 12 [10 - 14] | 12 [10 - 14] | <0.001 |
| **P/F Ratio ^b^** | 120 [88 - 173] | 145 [107 - 197] | 108 [81 - 148] | <0.001 |
| **ARDS severity ^b^** |  |  |  |  |
| **Mild** | 128 (15) | 76 (23) | 52 (10) | <0.001 |
| **Moderate** | 426 (50) | 183 (56) | 243 (46) |  |
| **Severe** | 298 (35) | 69 (21) | 229 (44) |  |
| **Tidal Volume (mL) ^b^** | 460 [420 - 500] | 470 [425 - 500] | 460 [420 - 500] | 0.186 |
| **Tidal Volume/PBW (mL/kg) ^b^** | 7.0 [6.3 - 7.8] | 7.1 [6.3 - 7.9] | 7 [6.2 - 7.8] | 0.140 |
| **Peak pressure (cmH_2_O) ^b^** | 30 [26 - 32] | 29 [26 - 32] | 30 [27 - 33] | 0.155 |
| **Plateau Pressure (cmH_2_O) ^b^** | 24 [22 - 27] | 24 [21 - 26] | 25 [22 - 28] | <0.001 |
| **Driving Pressure (cmH_2_O) ^b^** | 12 [9 - 14] | 12 [9 - 13] | 12 [9 - 14] | 0.120 |
| **RS Compliance (mL/cmH_2_O) ^b^** | 40 [33 - 50] | 42 [35 - 50] | 38 [32 - 50] | 0.035 |
| **pH** | 7.39 [7.32 - 7.46] | 7.40 [7.33 - 7.46] | 7.39 [7.31 - 7.45] | 0.220 |
| **PaO_2_ (mmHg)** | 80 [67 - 101] | 86 [70 - 108] | 77 [65 - 97] | <0.001 |
| **PaCO_2_ (mmHg)** | 43 [36 - 52] | 43 [37 - 51] | 43 [36 - 53] | 0.710 |
| **Ventilatory ratio** | 1.7 [1.4 - 2.2] | 1.7 [1.3 - 2.1] | 1.8 [1.4 - 2.2] | 0.061 |
| **Na^+^ (mEq/L)** | 138 [135 - 141] | 138 [135 - 141] | 138 [135 - 141] | 0.850 |
| **K^+^ (mEq/L)** | 3.9 [3.6 - 4.2] | 3.9 [3.6 - 4.3] | 3.9 [3.5 - 4.2] | 0.036 |
| **Cl^-^ (mEq/L)** | 104 [101 - 107] | 104 [101 - 107] | 104 [101 - 106] | 0.327 |
| **Lactate (mmol/L)** | 1.2 [1 - 1.6] | 1.2 [0.9 - 1.6] | 1.2 [1 - 1.6] | 0.042 |
| **HCO3^-^ (mEq/L)** | 25.5 [23.2 - 27.9] | 25.6 [22.9 - 28] | 25.5 [23.2 - 27.8] | 0.901 |
| **BE (mEq/L)** | 0.6 [-2.1 - 3.2] | 0.7 [-2.4 - 3.3] | 0.5 [-2 - 3.2] | 0.955 |
| **Total WBC (x 10^3^/mm^3^)** | 8.9 [6.4 - 12.2] | 8.8 [6.4 - 11.9] | 8.9 [6.4 - 12.3] | 0.863 |
| **Neutrophils (x 10^3^/mm^3^)** | 7.4 [5.2 - 10.7] | 7.1 [4.7 - 10.2] | 7.6 [5.4 - 11] | 0.100 |
| **Lymphocytes (x 10^3^/mm^3^)** | 0.7 [0.5 - 1] | 0.7 [0.5 - 1] | 0.7 [0.4 - 1] | 0.036 |
| **Platelets (x 10^3^/mm^3^)** | 234 [178 - 307] | 245 [182 - 312] | 230 [177 - 303] | 0.232 |
| **Hb (g/dL)** | 12.3 [11 - 13.4] | 12 [10.6 - 13.2] | 12.4 [11.3 - 13.4] | 0.001 |
| **Hct (%)** | 36.5 [33.4 - 39.6] | 35.7 [31.4 - 39.2] | 36.8 [34.1 - 40.1] | 0.002 |
| **Creatinine (mg/dL)** | 0.89 [0.7 - 1.11] | 0.88 [0.7 - 1.13] | 0.9 [0.7 - 1.1] | 0.753 |
| **Bilirubin (mg/dL)** | 0.7 [0.5 - 1] | 0.7 [0.5 - 1.1] | 0.7 [0.5 - 1] | 0.388 |
| **C-RP (mg/dL)** | 14.3 [5.9 - 22.7] | 14.2 [5.7 - 22.6] | 14.4 [6.3 - 22.8] | 0.401 |
| **PCT (ng/mL)** | 0.4 [0.2 - 1.2] | 0.3 [0.2 - 1.1] | 0.4 [0.2 - 1.2] | 0.327 |
| **LDH (units/L)** | 479 [359 - 640] | 424 [324 - 593] | 507 [392 - 667] | <0.001 |
| **D-dimer (ng/mL)** | 1492 [608 - 4602] | 1190 [520 - 3470] | 1730 [690 - 6576] | 0.001 |
| **Ferritin (ng/mL)** | 1408 [811 - 2399] | 1214 [668 - 1903] | 1552 [1031 - 2491] | 0.003 |
| **Heart Rate (bpm)** | 85 [72 - 96] | 80 [70 - 95] | 85 [75 - 96] | 0.002 |
| **Mean Arterial Pressure (mmHg)** | 85 [73 - 95] | 83 [73 - 95] | 85 [75 - 95] | 0.200 |

Data are presented as median [interquartile range] or Frequency (percentage). BMI = Body Mass Index; SOFA: Sequential Organ Failure Assessment; SAPS II: Simplified Acute Physiology Score II; APACHE II: Acute Physiologic Assessment and Chronic Health Evaluation II; IMV: Invasive Mechanical Ventilation; FiO_2_: Inspired fraction of oxygen; PEEP: Positive End-Expiratory Pressure; P/F: ratio between PaO_2_ and FiO_2_; ARDS: Acute Respiratory Distress Syndrome; PBW: Predicted Body Weight; PaO_2_: partial pressure of oxygen in arterial blood; PaCO_2_: partial pressure of carbon dioxide in arterial blood; BE: Base Excess; WBC: White blood Cells; Hb: Hemoglobin; Hct: Hematocrit; C-RP: C-Reactive Protein; PCT: Procalcitonin; LDH: lactate dehydrogenase; LOS = Length of stay; ^a^ Patients intubated same day of ICU admission; ^b^ values refer to patients intubated on the same day of ICU admission

**Table E3. Complete patients' characteristics at admission in ICU and outcome divided by Responders vs Non-Responders**

| **Variables** | **Overall**  **(n = 78)** | **O_2_-Non-responders**  **(n = 17)** | **O_2_-Responders**  **(n = 61)** | **p-value** |
| --- | --- | --- | --- | --- |
| **Males, no. (%)** | 61 (78) | 13 (77) | 48 (79) | 0.845 |
| **Age (years)** | 62 [51 - 68] | 56 [51 - 66] | 62 [52 - 68] | 0.389 |
| **BMI (kg/m^2^)** | 27 [25 - 31] | 26 [24 - 31] | 27 [26 - 31] | 0.670 |
| **Weight (kg)** | 83 [73 - 90] | 77 [70 - 90] | 85 [74 - 90] | 0.593 |
| **Height (cm)** | 170 [166 - 175] | 175 [165 - 175] | 170 [170 - 175] | 0.892 |
| **Charlson's Comorbidity Index** | 2 [1 - 3] | 2 [1 - 3] | 2 [1 - 3] | 0.429 |
| **Comorbidities** |  |  |  |  |
| Hypertension, no. (%) | 39 (50) | 7 (41) | 32 (53) | 0.411 |
| Others cardiovascular, no. (%) | 10 (13) | 2 (12) | 8 (13) | 0.883 |
| Diabetes, no. (%) | 8 (10) | 3 (18) | 5 ( 8) | 0.256 |
| Respiratory, no. (%) | 9 (12) | 1 ( 6) | 8 (13) | 0.409 |
| Malignancy, no. (%) | 3 ( 4) | 0 ( 0) | 3 ( 5) | 0.351 |
| Immunological, no. (%) | 2 ( 3) | 0 ( 0) | 2 ( 3) | 0.449 |
| Hepatic, no. (%) | 2 ( 3) | 0 ( 0) | 2 ( 3) | 0.449 |
| Renal, no. (%) | 0 (0) | 0 (0) | 0 (0) | NA |
| Others, no. (%) | 40 (51) | 9 (53) | 31 (51) | 0.877 |
| None, no. (%) | 20 (26) | 5 (29) | 15 (25) | 0.687 |
| **SOFA Score** | 4 [3 - 5] | 4 [3 - 5] | 4 [4 - 5] | 0.294 |
| **SAPS II Score** | 42 [35 - 48] | 41 [36 - 42] | 44 [35 - 49] | 0.260 |
| **APACHE II Score** | 10 [8 - 12] | 11 [8 - 14] | 10 [8 - 12] | 0.620 |
| **Temperature (°C)** | 37 [36.1 - 37.7] | 36.8 [36.4 - 37.9] | 37 [36 - 37.6] | 0.823 |
| **Symptom onset to admission** | 8 [6 – 12] | 9 [7 – 16] | 8 [6 – 12] | 0.408 |
| **Symptom onset to intubation** | 8.5 [6 – 11.5] | 9 [6- 16] | 8 [6 – 11] | 0.492 |
| **Presenting symptoms** |  |  |  |  |
| **Fever** | 71 (91) | 15 (88) | 56 (92) | 0.649 |
| **Dyspnea** | 50 (64) | 14 (82) | 36 (59) | 0.076 |
| **Cough** | 36 (46) | 8 (47) | 28 (46) | 0.933 |
| **Asthenia** | 9 (12) | 3 (18) | 6 ( 10) | 0.373 |
| **Myalgias** | 0 (0) | 0 (0) | 0 (0) | NA |
| **Headache** | 2 ( 3) | 0 ( 0) | 2 ( 3) | 0.449 |
| **Respiratory Failure** | 8 (10) | 2 (12) | 6 (10) | 0.817 |
| **Oxygen delivery before IMV, no. (%)** | 74 (99) | 17 (100) | 57 (98) | 0.586 |
| **Oxygen delivery time before IMV (hours)** | 42 [14 - 65] | 42 [24 - 65] | 42 [12 - 66] | 0.496 |
| **Intubated, no. (%) ^a^** | 75 (96) | 16 (94) | 59 (97) | 0.622 |
| **Respiratory Rate (breaths/min) ^b^** | 20 [18 - 22] | 19 [18 - 22] | 20 [17 - 22] | 0.813 |
| **FiO2 (%) ^b^** | 75 [60 - 90] | 80 [60 - 88] | 70 [60 - 90] | 0.839 |
| **PEEP (cmH_2_O) ^b^** | 14 [12 - 15] | 14 [11 - 14] | 14 [12 - 15] | 0.662 |
| **PaO_2_/F_I_O_2_ ratio  ^b^** | 111 [83 - 164] | 99 [72 - 150] | 114 [85 - 168] | 0.419 |
| **ARDS severity, no. (%) ^b^** |  |  |  |  |
| **Mild** | 10 (13) | 2 (13) | 8 (14) | 0.721 |
| **Moderate** | 34 (45) | 6 (38) | 28 (48) |  |
| **Severe** | 31 (41) | 8 (50) | 23 (39) |  |
| **Tidal Volume/PBW (mL/kg)  ^b^** | 7.0 [6.4 - 7.8] | 7.2 [6.2 - 7.9] | 7.0 [6.4 - 7.8] | 0.707 |
| **Plateau Pressure (cmH_2_O)  ^b^** | 25 [22 - 27] | 27 [24 - 28] | 24 [22 - 27] | 0.043 |
| **Driving Pressure (cmH_2_O)  ^b^** | 12 [9 - 14] | 14 [12 - 15] | 12 [8 - 13] | 0.022 |
| **Respiratory System Compliance (mL/cmH_2_O) ^b^** | 42 [32 - 53] | 34 [30 - 45] | 45 [34 - 56] | 0.018 |
| **pH** | 7.36 [7.31 - 7.41] | 7.33 [7.29 - 7.42] | 7.37 [7.32 - 7.41] | 0.244 |
| **PaO_2_ (mmHg)** | 78 [70 - 95] | 76 [68 - 90] | 80 [70 - 99] | 0.443 |
| **PaCO_2_ (mmHg)** | 47 [40 - 56] | 50 [39 - 58] | 47 [41 - 55] | 0.417 |
| **Ventilatory ratio** | 1.8 [1.4 – 2.0] | 1.8 [1.4 – 2.5] | 1.8 [1.4 – 2.0] | 0.345 |
| **Na^+^ (mEq/L)** | 138 [135 - 140] | 137 [133 - 140] | 138 [136 - 140] | 0.280 |
| **K^+^ (mEq/L)** | 3.9 [3.5 - 4.2] | 3.7 [3.4 - 4] | 3.9 [3.6 - 4.2] | 0.438 |
| **Cl^-^ (mEq/L)** | 104 [101 - 108] | 103 [101 - 105] | 105 [101 - 108] | 0.273 |
| **Lactate (mmol/L)** | 1.2 [1 - 1.5] | 1.1 [1 - 1.3] | 1.2 [1 - 1.6] | 0.427 |
| **HCO3^-^ (mEq/L)** | 25.7 [23.4 - 28.4] | 26.1 [24.6 - 27.5] | 25.7 [23.3 - 28.6] | 0.326 |
| **BE (mEq/L)** | 1 [-1.7 - 3.1] | 0.9 [-1.2 - 3.2] | 1.1 [-2 - 3] | 0.850 |
| **Total WBC (x 10^3^/mm^3^)** | 9.3 [7.1 - 13] | 8.7 [7.2 - 11.2] | 9.8 [7 - 13.4] | 0.574 |
| **Neutrophils (x 10^3^/mm^3^)** | 8.1 [6 - 11.7] | 7.3 [5.7 - 9.6] | 8.6 [6 - 12] | 0.316 |
| **Lymphocytes (x 10^3^/mm^3^)** | 0.7 [0.4 - 1] | 0.9 [0.5 - 1] | 0.7 [0.4 - 0.9] | 0.099 |
| **Platelets (x 10^3^/mm^3^)** | 226 [163 - 288] | 215 [180 - 301] | 226 [162 - 287] | 0.620 |
| **Hb (g/dL)** | 12.4 [11.4 - 13.4] | 12.1 [11.5 - 13.3] | 12.5 [11.4 - 13.5] | 0.284 |
| **Hct (%)** | 36.7 [34.3 - 40.2] | 36.6 [33.9 - 38.6] | 37.3 [34.8 - 40.4] | 0.077 |
| **Creatinine (mg/dL)** | 0.89 [0.7 - 1.11] | 0.8 [0.63 - 0.9] | 0.9 [0.7 - 1.16] | 0.049 |
| **Bilirubin (mg/dL)** | 0.7 [0.5 - 1.1] | 0.8 [0.5 - 1] | 0.7 [0.4 - 1.2] | 0.474 |
| **C-RP (mg/dL)** | 12.7 [5.8 - 18.9] | 13.4 [8.5 - 19.8] | 12 [5.6 - 18.9] | 0.610 |
| **PCT (ng/mL)** | 0.4 [0.2 - 0.8] | 0.2 [0.2 - 0.5] | 0.4 [0.2 - 0.9] | 0.291 |
| **LDH (units/L)** | 414 [307 - 490] | 418 [329 - 485] | 400 [301 - 490] | 0.620 |
| **D-dimer (ng/mL)** | 2064 [1261 - 7813] | 2811 [1385 - 7193] | 1716 [1214 - 8120] | 0.786 |
| **Ferritin (ng/mL)** | 1708 [1158 - 2122] | 1910 [1038 - 2539] | 1692 [1325 - 2051] | 0.974 |
| **Heart Rate (bpm)** | 80 [70 - 95] | 88 [71 - 109] | 80 [70 - 90] | 0.137 |
| **Mean Arterial Pressure (mmHg)** | 80 [70 - 90] | 78 [70 - 98] | 80 [70 - 88] | 0.871 |

Data are presented as median [interquartile range] or Frequency (percentage). BMI: Body Mass Index; SOFA: Sequential Organ Failure Assessment; SAPS II: Simplified Acute Physiology Score II; APACHE II: Acute Physiologic Assessment and Chronic Health Evaluation II; IMV: Invasive Mechanical Ventilation; FiO_2_: Inspired fraction of oxygen; PEEP: Positive End-Expiratory Pressure; P/F: ratio between PaO_2_ and FiO_2_; ARDS: Acute Respiratory Distress Syndrome; PBW: Predicted Body Weight; PaO_2_: partial pressure of oxygen in arterial blood; PaCO_2_: partial pressure of carbon dioxide in arterial blood; BE: Base Excess; WBC: White blood Cells; Hb: Hemoglobin; Hct: Hematocrit; C-RP: C-Reactive Protein; PCT: Procalcitonin; LDH: lactate dehydrogenase; LOS = Length of stay; ^a^ Patients intubated same day of ICU admission; ^b^ values refer to patients intubated on the same day of ICU admission

**Table E4. Complete patients' characteristics at admission in ICU and outcome divided by CO_2_-Responders vs CO_2_-Non-Responders**

| **Variable** | **Overall**  **(N=78)** | **CO_2_-Non-Responders**  **(N = 41, 53%)** | **CO_2_-Responders**  **(N= 37, 47%)** | **p-value** |
| --- | --- | --- | --- | --- |
| **Males, no. (%)** | 61 (78.21%) | 31 (75.61%) | 30 (81.08%) | 0.559 |
| **Age (years)** | 62 [51 - 68] | 56 [50 - 64] | 65 [59 - 70] | 0.005 |
| **BMI (kg/m2)** | 27 [25 - 31] | 27 [25 - 31] | 28 [26 - 31] | 0.451 |
| **Weight (kg)** | 83 [73 - 90] | 80 [70 - 90] | 85 [75 - 90] | 0.318 |
| **Height (cm)** | 170 [166 - 175] | 170 [168 - 175] | 170 [166 - 178] | 0.843 |
| **Charlson's Comorbidity Index** | 2 [1 - 3] | 2 [1 - 3] | 2 [2 - 3] | 0.076 |
| **Comorbidities** |  |  |  |  |
| Hypertension, no. (%) | 39 (50.00%) | 14 (34.15%) | 25 (67.57%) | 0.003 |
| Others cardiovascular, no. (%) | 10 (12.82%) | 4 ( 9.76%) | 6 (16.22%) | 0.394 |
| Diabetes, no. (%) | 8 (10.26%) | 4 ( 9.76%) | 4 (10.81%) | 0.878 |
| Respiratory, no. (%) | 9 (11.54%) | 3 ( 7.32%) | 6 (16.22%) | 0.219 |
| Malignancy, no. (%) | 3 ( 3.85%) | 1 ( 2.44%) | 2 ( 5.41%) | 0.496 |
| Immunological, no. (%) | 2 ( 2.56%) | 1 ( 2.44%) | 1 ( 2.70%) | 0.941 |
| Hepatic, no. (%) | 2 ( 2.56%) | 2 ( 4.88%) | 0 ( 0.00%) | 0.174 |
| Renal, no. (%) | 0 (0.0%) | 0 (0.0%) | 0 (0.0%) | . |
| Others, no. (%) | 40 (51.28%) | 18 (43.90%) | 22 (59.46%) | 0.17 |
| None, no. (%) | 20 (25.64%) | 17 (41.46%) | 3 ( 8.11%) | 0.001 |
| **SOFA Score** | 4 [3 - 5] | 4 [3 - 5] | 4 [4 - 5] | 0.879 |
| **SAPS II Score** | 42 [35 - 48] | 41 [34 - 44] | 45 [39 - 51] | 0.015 |
| **APACHE II Score** | 10 [8 - 12] | 10 [8 - 14] | 9 [8 - 12] | 0.371 |
| **Temperature (°C)** | 37 [36.1 - 37.7] | 37 [36.3 - 37.7] | 37 [36 - 37.7] | 0.56 |
| **Symptom onset to admission** | -8 [-12 - -6] | -8 [-11 - -6] | -8 [-13 - -6] | 0.676 |
| **Symptom onset to intubation** | -9 [-11.5 - -6] | -8 [-11 - -6] | -9 [-13 - -6] | 0.619 |
| **Fever** | 71 (91.03%) | 35 (85.37%) | 36 (97.30%) | 0.066 |
| **Dyspnea** | 50 (64.10%) | 26 (63.41%) | 24 (64.86%) | 0.894 |
| **Cough** | 36 (46.15%) | 18 (43.90%) | 18 (48.65%) | 0.675 |
| **Asthenia** | 9 (11.54%) | 6 (14.63%) | 3 ( 8.11%) | 0.368 |
| **Myalgias** | 0 (0.0%) | 0 (0.0%) | (0.0%) | . |
| **Headache** | 2 ( 2.56%) | 1 ( 2.44%) | 1 ( 2.70%) | 0.941 |
| **Respiratory Failure** | 8 (10.26%) | 4 ( 9.76%) | 4 (10.81%) | 0.878 |
| **Oxygen delivery time before IMV (hours)** | 42 [14 - 65] | 48 [12 - 72] | 24 [14 - 65] | 0.486 |
| **Oxygen delivery before IMV, no. (%)** | 74 (98.67%) | 39 (100.0%) | 35 (97.22%) | 0.295 |
| **Intubated, no. (%) ^a^** | 75 (96.15%) | 40 (97.56%) | 35 (94.59%) | 0.496 |
| **Respiratory Rate (breaths/min) ^b^** | 20 [18 - 22] | 20 [18 - 22] | 20 [18 - 22] | 0.666 |
| **FiO2 (%) ^b^** | 75 [60 - 90] | 75 [60 - 90] | 75 [60 - 80] | 0.326 |
| **PEEP (cmH2O) ^a^** | 14 [12 - 15] | 13 [11 - 15] | 14 [12 - 14] | 0.707 |
| **PaO2/FIO2 ratio b** | 111 [83 - 164] | 111 [81 - 166] | 117 [85 - 153] | 0.865 |
| **ARDS severity, no. (%) ^b^** |  |  |  |  |
| **Mild** | 10 (13.33%) | 6 (15.00%) | 4 (11.43%) | 0.836 |
| **Moderate** | 34 (45.33%) | 17 (42.50%) | 17 (48.57%) |  |
| **Severe** | 31 (41.33%) | 17 (42.50%) | 14 (40.00%) |  |
| **Tidal Volume/PBW (mL/kg) ^b^** | 7 [6.4 - 7.8] | 7.1 [6.4 - 7.7] | 6.8 [6.4 - 7.8] | 0.975 |
| **Plateau Pressure (cmH2O) ^b^** | 25 [22 - 27] | 25 [24 - 28] | 24 [22 - 27] | 0.341 |
| **Driving Pressure (cmH2O) ^b^** | 12 [9 - 14] | 12 [9 - 14] | 12 [8 - 14] | 0.34 |
| **Respiratory System Compliance (mL/cmH_2_O) ^b^** | 42 [32 - 53] | 40 [32 - 51] | 44 [32 - 56] | 0.413 |
| **pH** | 7.36 [7.31 - 7.41] | 7.34 [7.31 - 7.42] | 7.37 [7.315 - 7.405] | 0.717 |
| **PaO_2_ (mmHg)** | 78 [70 - 95] | 82 [73 - 101] | 77 [68 - 87] | 0.205 |
| **Ventilatory ratio** | 1.8 [1.4 – 2.0] | 1.8 [1.4 – 2.1] | 1.7 [1.5 – 2.0] | 0.778 |
| **PaCO_2_ (mmHg)** | 47 [40 - 56] | 48 [39 - 57] | 46 [41 - 53] | 0.314 |
| **Na^+^ (mEq/L)** | 138 [135 - 140] | 138 [135 - 141] | 138 [136 - 140] | 0.591 |
| **K^+^ (mEq/L)** | 3.9 [3.5 - 4.2] | 4 [3.6 - 4.2] | 3.8 [3.5 - 4.3] | 0.734 |
| **Cl^-^ (mEq/L)** | 104 [101 - 108] | 104 [101 - 108] | 105 [101 - 108] | 0.746 |
| **Lactate (mmol/L)** | 1.2 [1 - 1.5] | 1.1 [0.9 - 1.5] | 1.2 [1.1 - 1.6] | 0.136 |
| **HCO_3_^-^ (mEq/L)** | 25.7 [23.4 - 28.4] | 26 [23.6 - 28.2] | 25.7 [23.1 - 28.7] | 0.839 |
| **BE (mEq/L)** | 1 [-1.7 - 3.1] | 0.9 [-1.2 - 3] | 1.1 [-2 - 3.3] | 0.725 |
| **Total WBC (x 10^3^/mm^3^)** | 9.3 [7.1 - 13] | 9.8 [7.7 - 13.6] | 8.4 [6.7 - 12.1] | 0.141 |
| **Neutrophils (x 10^3^/mm^3^)** | 8.1 [6 - 11.7] | 8.9 [6.1 - 12] | 7.3 [5.8 - 10.6] | 0.324 |
| **Lymphocytes (x 10^3^/mm^3^)** | 0.7 [0.4 - 1] | 0.7 [0.4 - 1] | 0.7 [0.4 - 0.8] | 0.373 |
| **Platelets (x 10^3^/mm^3^)** | 226 [163 - 288] | 235 [160 - 301] | 211 [172 - 251] | 0.525 |
| **Hb (g/dL)** | 12.4 [11.4 - 13.4] | 12.3 [11.3 - 13.2] | 12.6 [12.1 - 13.8] | 0.068 |
| **Hct (%)** | 36.7 [34.3 - 40.2] | 36.3 [33.6 - 38.4] | 38.6 [35.1 - 41.3] | 0.024 |
| **Creatinine (mg/dL)** | 0.89 [0.7 - 1.11] | 0.9 [0.69 - 1.09] | 0.85 [0.7 - 1.14] | 0.715 |
| **Bilirubin (mg/dL)** | 0.7 [0.5 - 1.1] | 0.6 [0.4 - 0.9] | 0.7 [0.5 - 1.3] | 0.113 |
| **C-RP (mg/dL)** | 12.7 [5.8 - 18.9] | 15.4 [8 - 20.4] | 9.6 [5 - 18] | 0.176 |
| **PCT (ng/mL)** | 0.4 [0.2 - 0.8] | 0.4 [0.2 - 0.9] | 0.3 [0.1 - 0.7] | 0.224 |
| **LDH (units/L)** | 414 [307 - 490] | 422 [321 - 526] | 397 [297 - 453] | 0.121 |
| **D-dimer (ng/mL)** | 2064 [1261 - 7813] | 2167 [1214 - 8770] | 1960 [1338 - 6134] | 0.697 |
| **Ferritin (ng/mL)** | 1708 [1158 - 2122] | 1788 [1241 - 2581] | 1396 [1060 - 1920] | 0.286 |
| **Heart Rate (bpm)** | 80 [70 - 95] | 82 [70 - 100] | 80 [70 - 90] | 0.172 |
| **Mean Arterial Pressure (mmHg)** | 80 [70 - 90] | 85 [70 - 94] | 77 [70 - 87] | 0.187 |
| **ICU mortality, no. (%)** | 34 (43.59%) | 15 (36.59%) | 19 (51.35%) | 0.189 |
| **Hospital mortality, no. (%)** | 34 (43.59%) | 15 (36.59%) | 19 (51.35%) | 0.189 |
| **ICU LOS (days)** | 18 [11 - 34] | 18 [12 - 45] | 19 [10 - 31] | 0.423 |
| **Hospital LOS (days)** | 36 [17 - 58] | 35 [22 - 67] | 39 [13 - 55] | 0.512 |
| **Mechanical Ventilation (days)** | 19 [11 - 35] | 18 [12 - 35] | 19 [10 - 35] | 0.627 |

Data are presented as median [interquartile range] or Frequency (percentage). BMI: Body Mass Index; SOFA: Sequential Organ Failure Assessment; SAPS II: Simplified Acute Physiology Score II; APACHE II: Acute Physiologic Assessment and Chronic Health Evaluation II; IMV: Invasive Mechanical Ventilation; FiO_2_: Inspired fraction of oxygen; PEEP: Positive End-Expiratory Pressure; P/F: ratio between PaO_2_ and FiO_2_; ARDS: Acute Respiratory Distress Syndrome; PBW: Predicted Body Weight; PaO_2_: partial pressure of oxygen in arterial blood; PaCO_2_: partial pressure of carbon dioxide in arterial blood; BE: Base Excess; WBC: White blood Cells; Hb: Hemoglobin; Hct: Hematocrit; C-RP: C-Reactive Protein; PCT: Procalcitonin; LDH: lactate dehydrogenase; LOS = Length of stay; ^a^ Patients intubated same day of ICU admission; ^b^ values refer to patients intubated on the same day of ICU admission

**Figure E2 - Variation in PaO_2_/FiO_2_ ratio with prone position when dividing the overall population in tertiles of pre-pronation driving pressure**

Figure shows median, 10^th^, 25^th^, 75th and 90^th^ percentiles as vertical boxes with error bars, black dots represent outliers, grey dashed lines represent mean. Grey boxes represent PaO2/FiO2 difference (prone position value – baseline value); white boxes represent pre-pronation Driving Pressure as P = 0.010 ANOVA on Ranks;

* P<0.05 vs 1st tertile; § P<0.05 vs 2nd tertile.
